# Supplementary material for: Spectral organ fingerprints for machine learning-based intraoperative tissue classification with hyperspectral imaging in a porcine model
Source: Sci Rep. 2022 Jun 30;12:11028. doi: 10.1038/s41598-022-15040-w (PMC9247052; doi:10.1038/s41598-022-15040-w)
Supplement: Supplementary file 1 — Supplementary Information. [file 41598_2022_15040_MOESM1_ESM.docx]

**Supplement**

**Spectral organ fingerprints for machine learning-based intraoperative tissue classification with hyperspectral imaging in a porcine model**

Studier-Fischer, A.^1^, Seidlitz, S.^2,3^, Sellner, J.^2,3,4^, Özdemir, B.^1^, Wiesenfarth, M.^5^, Ayala, L.^2^, Odenthal, J.^1^, Knödler, S.^1^, Kowalewski, K.F.^6^, Haney, C.M.^1^, Camplisson, I.^7^, Dietrich, M.^8^, Schmidt, K.^9^, Salg, G.A.^1^, Kenngott, H.G.^1^, Adler, T.J.^2,10^, Schreck, N.^5^, Kopp-Schneider, A.^5^, Maier-Hein, K.^2,3,4^, Maier-Hein, L. ^2,3,4,10^, Müller-Stich, B.P.^1^, Nickel, F.*^1,3^

**Supplementary Text 1: Data overview and standardized recordings protocol**

The complete dataset for this study consists of 9,059 images of 46 pigs with a total of 17,777 annotations for 20 different organ classes. The exact data distribution can be seen in **Supplementary Figure 1**. It is important to note that one recording can have multiple organs in its field of view and therefore can have multiple organs annotated. Hence, the total number of annotations across all organs is greater than the total number of images in the data set. Within one organ, the total number of recordings equals the total number of annotations. Every image has at least one organ annotation. The number of recordings per animal is heterogeneous since the number of “standardized” images recorded for pigs P36 to P46 is generally higher than the number of images recorded when imitating intraoperative reality. Furthermore, the distribution of number of images per organ is heterogeneous since some organs naturally occur more often in the field of view of the camera, e.g. during recordings of the gallbladder, the liver is always in the same field of view but for liver recordings, the gallbladder is not always visible. The linear mixed models analysis was done on the recordings marked with scratched boxes in **Supplementary Figure 1**. Testing of the machine learning algorithm was performed on all images of the 8 animals marked with asterisks.


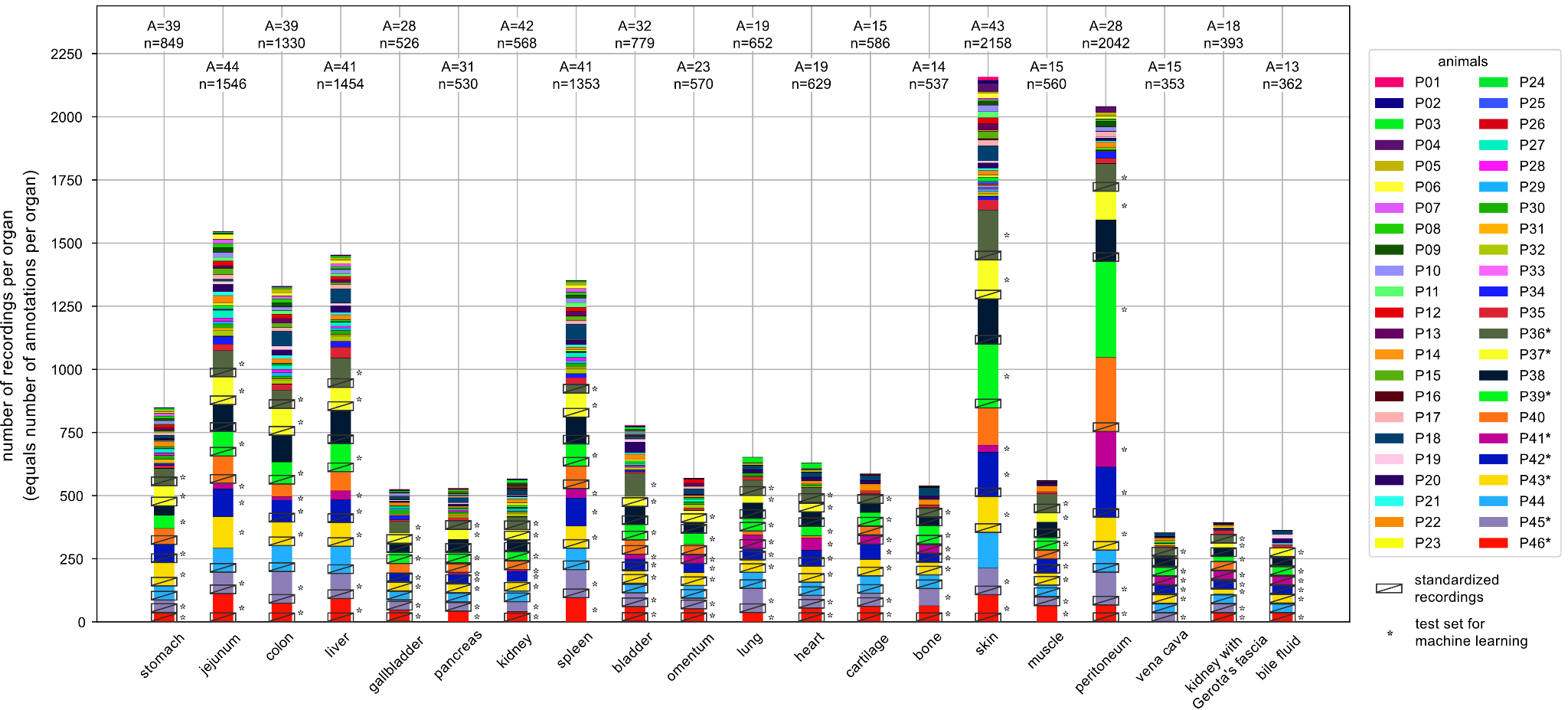


**Supplementary Figure 1 | Database visualization.** One color corresponds to one pig with P01 at the top and P46 at the bottom of the bar chart. The scratched box indicates the recordings from the 11 standardized pig measurements that were used for the linear mixed models analysis with 36 recordings per box and 8 boxes per organ. The asterisk indicates the 8 pigs that were used as a test set and for reporting accuracy of the machine learning algorithm.

The standardized images were recorded with a specific protocol, which is illustrated in **Supplementary Figure 2** including 3 repetitions in 3 angles in 4 situs of each organ (for 8 pigs per organ) in a total of 11 pigs.


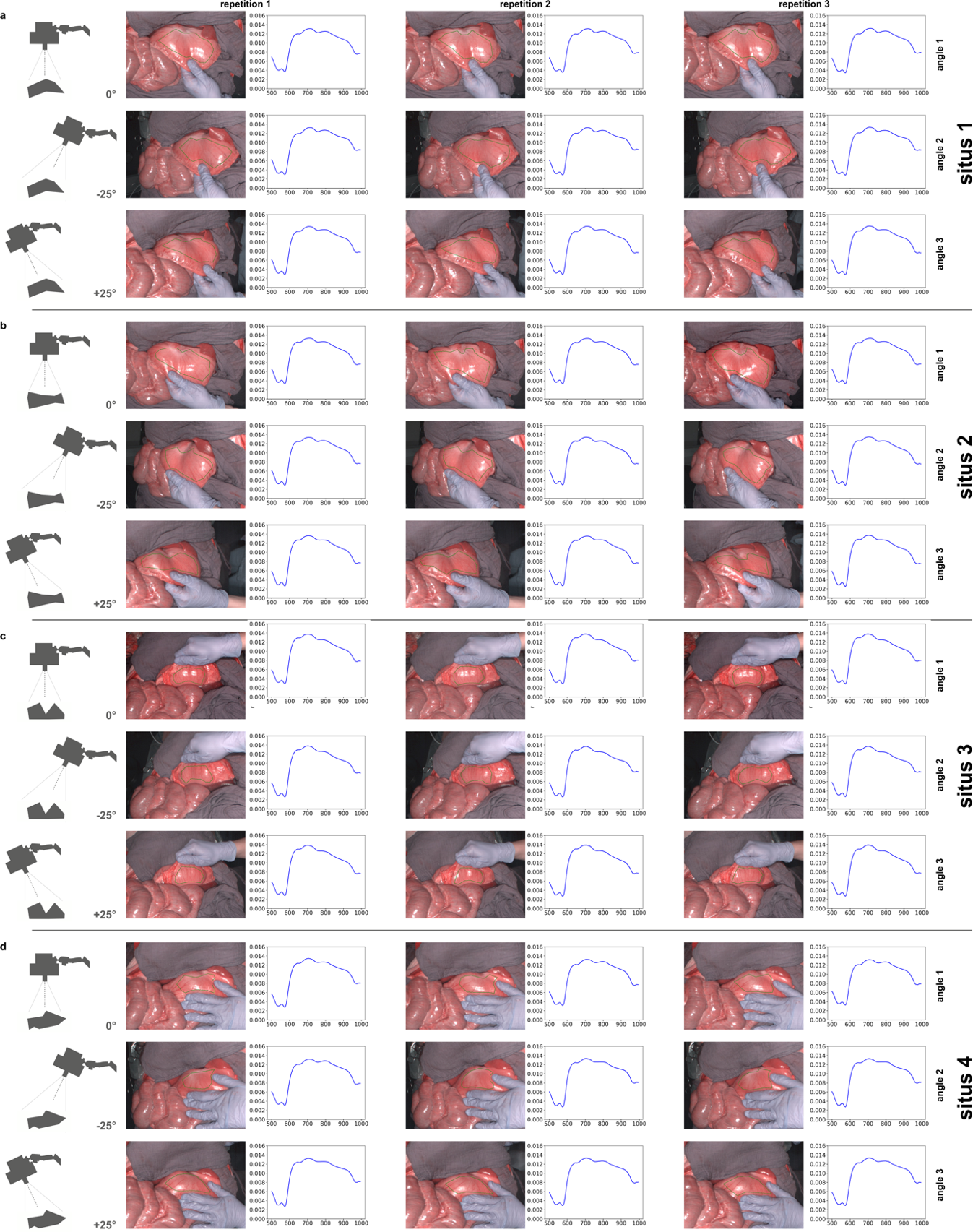


**Supplementary Figure 2 | Schematic recordings protocol for the standardized dataset.** The schematic visualization depicts the 36 recordings that were taken per organ in 8 animals. These include 3 repetitions in 3 angles in 4 situs. A stomach recording is used as an example together with the extracted reflectance spectrum of the corresponding annotation.

**Supplementary Text 2: Structured model analysis**

In order to enhance our understanding of the single influencing components that make up the recorded spectra, the complete dataset was systematically deconstructed and consecutively reconstructed through a structured model ^1^. For this, an additive model was fitted regressing reflectance (assuming a Gaussian distribution) on a smooth overall mean curve along wavelength plus smooth deviations from this curve by organs plus a smooth random effect for “pig” and a random intercept for “image” as well as a fixed effect for “angle” was calculated (**Supplementary Figure 3**). P-splines with 25 knots were used for estimation of smooth terms, except for smooth random pig effects where 10 knots were used. First-order penalty was used for smooth organ-specific deviations and quadratic penalties otherwise ^2^. The high similarity between raw data and its prediction indicates how the entire dataset has clear components that contribute to the final shape of reflectance.

The structured model provides a different view on hyperspectral tissue characterizations of organs shown in **Figure 1** by decomposing the wavelength-reflectance curves into an overall mean curve shared by all organs (a) and organ-specific deviations from this overall mean curve (d) as well as smooth pig-specific deviations from the overall mean modelled as random effects (b) (**Supplementary Figure 3**). The organ-specific deviations in (d) can be further decomposed into a general mean level shift from the overall mean constant across wavelengths (c top) and differences in the shape of the organ-specific curves (c bottom). Panel (d) illustrates that reflectance curves were relatively constant for each organ across wavelengths above a wavelength of approximately 650 nm and below 900nm except for spleen and bile fluid. Panel (c top) shows which organs shared a similar mean reflectance level across all wavelengths. Bile fluid was intuitively shown to have a very different reflectance profile than the solid organs most strikingly visible in (c bottom). Panel (b) illustrates that after the organ effect was controlled for, the pig-specific variation of reflectance was relatively small across wavelengths up to 900 nm, (with curves between approximately -0.0005 and 0.0005) as compared to differences between organs with organ-specific deviations from the overall mean between +/- 0.004. Angle effects were small with -6.3e-06 (SE 1.9e-06) for 25° from body-left and 7.6e-06 (SE 1.9e-06) for 25° from body-right. 95 % of predicted image-specific deviations (image random effects) range between +/- 1e-08, reflecting the same finding as in the mixed model analysis that image is the second strongest effect and potentially more important than pig. Panel (e) illustrates that the model is suitable to predict reflectance relationships.


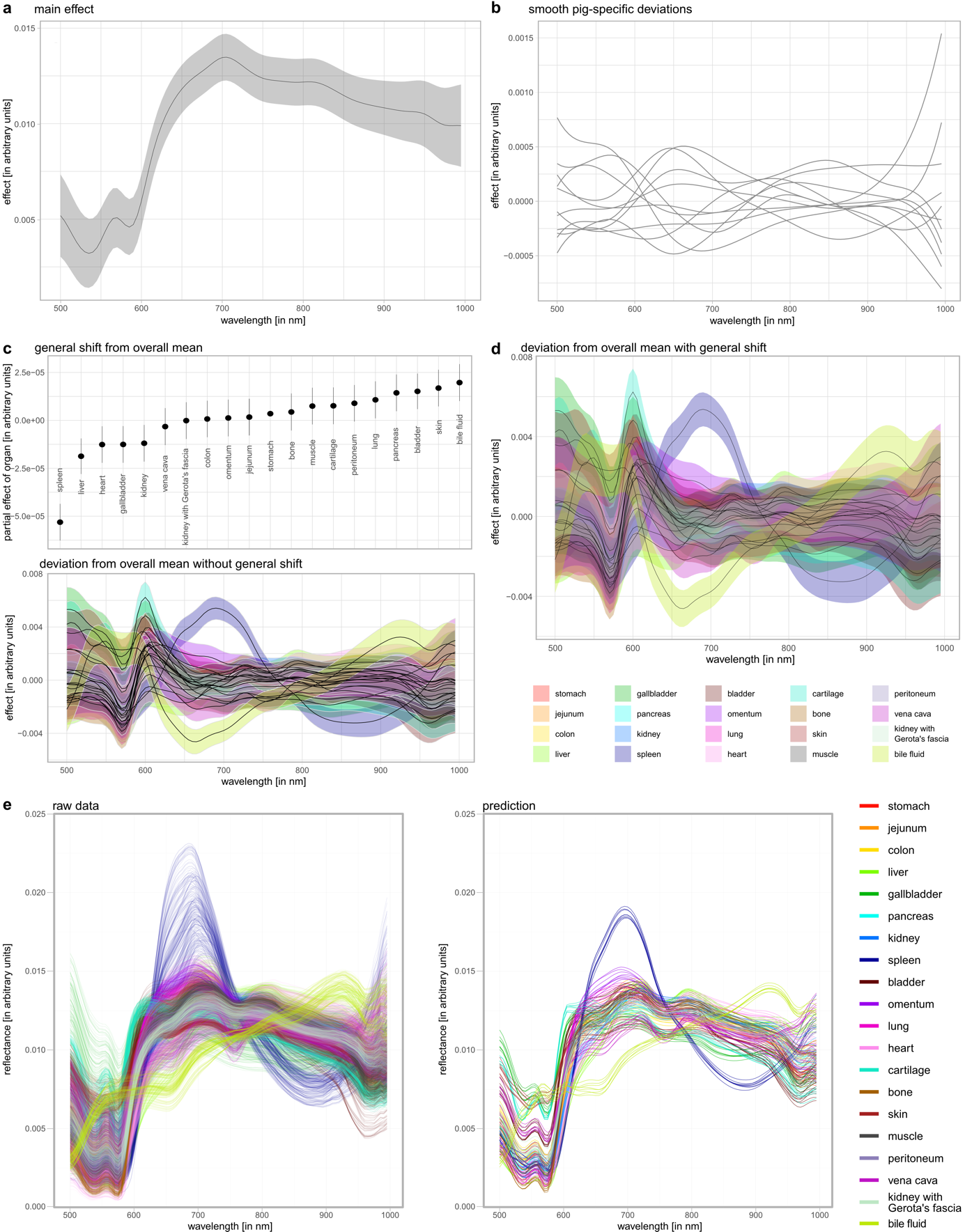


**Supplementary Figure 3 | Structured model.** **a**, Overall effect: displays the overall mean wavelength-reflectance relationship shared by all. **b**, pig random smooth effect independent of image and organ and overall mean. **c**, organ level shift (displays the level shift from the overall mean reflectance relationship for each organ) and deviation from overall mean (displays the deviation from the overall mean reflectance relationship for each organ; excluding level shift). **d**, organ-specific deviation from overall mean including level shift. Angle and image effects were not visualized as they can be sufficiently described in the text. **a-d**: 95 % pointwise confidence bands i.e. asymptotic pointwise confidence intervals based on normal distribution. **e**, raw data (on annotation level with one curve for every annotation of every organ in every pig) and prediction (on image level with one curve for every image of every organ in every pig) from the model.

**Supplementary Text 3: Quantification of organ specificity**

The sum of variances across components other than the factor “organ” (**Figure 4**) averaged across wavelengths can be used as an indicator for the spectral organ-specificity of the respective organ or tissue class. Organs with lower values have more organ-characteristic spectral signatures across observations and vice versa (**Supplementary Table 1**).

***Supplementary Table 1* | Explained standard deviation of reflectance for factors "pig", "angle", "image" and "repetition" and “together”, averaged across wavelengths**. “Together” is the explained standard deviation cumulatively for factors other than “organ”. It is not identical to the mathematical sum of the 4 factors as it is calculated by the root of the sum of variances of the 4 factors. A smaller “together” number indicates a more organ-characteristic spectrum. Table is sorted by increasing “together” values.

| ***organ*** | ***pig*** | ***image*** | ***angle*** | ***repetition*** | ***together*** |
| --- | --- | --- | --- | --- | --- |
| *pancreas* | 0.0002372 | 0.0002197 | 0.0000119 | 0.0000587 | 0.0003288 |
| *muscle* | 0.0002675 | 0.0002007 | 0.0000873 | 0.0000402 | 0.0003480 |
| *skin* | 0.0002958 | 0.0001978 | 0.0000294 | 0.0000449 | 0.0003598 |
| *stomach* | 0.0002710 | 0.0002178 | 0.0000805 | 0.0000541 | 0.0003610 |
| *omentum* | 0.0003353 | 0.0001515 | 0.0000679 | 0.0000503 | 0.0003775 |
| *colon* | 0.0002923 | 0.0002508 | 0.0000415 | 0.0000507 | 0.0003907 |
| *lung* | 0.0003995 | 0.0002310 | 0.0000000 | 0.0000426 | 0.0004628 |
| *kidney* | 0.0002861 | 0.0003590 | 0.0000877 | 0.0000583 | 0.0004710 |
| *kidney with Gerota’s fascia* | 0.0002389 | 0.0004025 | 0.0001051 | 0.0000551 | 0.0004829 |
| *jejunum* | 0.0003296 | 0.0003547 | 0.0000000 | 0.0000563 | 0.0004870 |
| *peritoneum* | 0.0003922 | 0.0002841 | 0.0000735 | 0.0000623 | 0.0004938 |
| *bone* | 0.0003622 | 0.0003105 | 0.0001440 | 0.0000811 | 0.0005049 |
| *bile fluid* | 0.0004762 | 0.0002321 | 0.0000000 | 0.0000472 | 0.0005313 |
| *cartilage* | 0.0005151 | 0.0003580 | 0.0000374 | 0.0000779 | 0.0006332 |
| *heart* | 0.0006199 | 0.0002700 | 0.0000408 | 0.0001097 | 0.0006862 |
| *bladder* | 0.0006353 | 0.0003516 | 0.0000072 | 0.0000543 | 0.0007282 |
| *vena cava* | 0.0006774 | 0.0003765 | 0.0000369 | 0.0001395 | 0.0007883 |
| *liver* | 0.0006620 | 0.0004690 | 0.0000627 | 0.0000731 | 0.0008170 |
| *gallbladder* | 0.0010187 | 0.0004690 | 0.0000980 | 0.0001061 | 0.0011307 |
| *spleen* | 0.0009827 | 0.0006778 | 0.0002601 | 0.0001160 | 0.0012272 |

**Supplementary Text 4: Annotation strategy**

Annotations were done on RGB images reconstructed from the HSI data, using the HyperGUI tool (<https://github.com/MIC-Surgery-Heidelberg/HyperGUI2.0_lite>). Possible labels were: “stomach”, “jejunum”, “colon”, “liver”, “gallbladder”, “pancreas”, “kidney”, “spleen”, “bladder”, “omentum”, “lung”, “heart”, “cartilage”, “bone”, “skin”, “muscle”, “peritoneum”, “vena cava”, “kidney with Gerota’s fascia” and “bile fluid”.

Non-semantic annotation was performed with a multi-point selection tool. Polygon areas were selected by omitting any areas with artefacts including tissue kinking, shade from the illumination, marginal areas, superficial blood vessels and fat, contamination with dyes or body fluids such as bile fluid, previous manipulation such as contusion or abrasion and possible impairment of perfusion such as thrombosis.

The regions were selected with the aim of including only highly representative areas; therefore, it is guaranteed that analyzed pixels were always 100% representative of the label. Consequently, there are additional adjacent pixels that could have been selected as well, but were not based on the judgement of the annotator and the premise to not include faulty or non-representative pixels under any circumstance. In case of several possible regions that were separated by aforementioned artefacts, the largest and most representative area was selected.

1 Wood, S. N. Fast stable restricted maximum likelihood and marginal likelihood estimation of semiparametric generalized linear models. *Journal of the Royal Statistical Society: Series B (Statistical Methodology)* **73**, 3-36, doi:<https://doi.org/10.1111/j.1467-9868.2010.00749.x> (2011).

2 Pedersen​, E. J., Miller, D. L., Simpson, G. L. & Ross, N. Hierarchical generalized additive models in ecology: an introduction with mgcv. *PeerJ 7:e6876*, doi:<https://doi.org/10.7717/peerj.6876> (2019).
